# Association among oral symptoms, oral health-related quality of life, and health-related quality of life in a sample of adults living with HIV/AIDS in Malaysia

**DOI:** 10.1186/s12903-017-0409-y

**Published:** 2017-08-22

**Authors:** Nurulasmak Mohamed, Norkhafizah Saddki, Azizah Yusoff, Anilawati Mat Jelani

**Affiliations:** 1 School of Dental Sciences, Universiti Sains Malaysia, Health Campus, 16150 Kubang Kerian, Kelantan Malaysia; 2 Department of Medicine, Hospital Raja Perempuan Zainab II, 15586 Kota Bharu, Kelantan Malaysia

**Keywords:** HIV, Quality of life, Oral health, Malaysia

## Abstract

**Background:**

Health-related quality of life (HRQOL) is a multidimensional construct that refers to an individual’s self-perceived well-being. This study used the revised Wilson and Cleary HRQOL model to investigate the associations among oral symptoms, oral health-related quality of life (OHRQOL), and HRQOL of people living with HIV/AIDS (PLWHA) in Malaysia.

**Methods:**

A total of 121 PLWHA receiving medical care in Kota Bharu (Kelantan, Malaysia) participated in this cross-sectional study. The Malay version of the short Oral Health Impact Profile (S-OHIP(M)) and the Malay version of the 36-item Medical Outcome Study Short Form (SF-36) were used to assess OHRQOL and HRQOL, respectively. A higher S-OHIP(M) score indicates greater oral impact and worse OHRQOL; a higher SF-36 score indicates better HRQOL. An additional structured self-administered questionnaire was used to obtain other variables of interest from the participants.

**Results:**

Most participants had at least one oral symptom (69.4%), and the most common oral symptom was a cavitated tooth (55.4%). The prevalence of oral impacts was 33.9%, and the mean S-OHIP(M) score was 8.8 (SD = 7.92). The mean S-OHIP(M) score was significantly higher in participants who had toothaches, cavitated teeth, gum abscesses, and bad breath. In addition, participants with lower S-OHIP(M) scores had significantly higher scores in all SF-36 domains.

**Conclusions:**

Our study provides evidence for an association among oral symptoms, OHRQOL, and HRQOL in PLWHA from Malaysia. In particular, the presence of oral symptoms was significantly associated with more severe oral impacts and poorer OHRQOL. The presence of less severe oral impacts was associated with a better HRQOL.

**Electronic supplementary material:**

The online version of this article (doi:10.1186/s12903-017-0409-y) contains supplementary material, which is available to authorized users.

## Background

HIV infection remains one of the world’s major public health issues. The use of highly active antiretroviral therapy (HAART) has reduced global AIDS-related deaths from 1.5 million in 2010 to 1.1 million in 2015 [1]. Additionally, sustained viral suppression and subsequent reduction of viral load in the blood and other bodily fluids has led to substantial decreases in the incidence of AIDS-defining events and AIDS-related morbidity, and has also reduced the number of new HIV infections because of the lower likelihood of HIV transmission per exposure event [2–5]. However, the number of new HIV infections being reported each year is still high, and the improved survival time means there is a persistent increase in the number of people living with chronic HIV infections [6]. Approximately 36.7 million people worldwide had HIV infections in 2015, compared to 33.3 million in 2010 [1]. Similarly, in Malaysia, there has been a steady decline in the annual number of new HIV cases and AIDS-related deaths since 2002 [7]. In 2014, Malaysia had an estimated 86,324 people living with HIV/AIDS (PLWHA) [7].

In tandem with the increased life expectancy of PLWHA, assessment of health-related quality of life (HRQOL) has become an important part of patient management [8]. The term HRQOL refers to an individual’s self-perceived well-being at any given period that contributes to satisfaction and happiness in life [9]. The HRQOL is a multidimensional construct, and previous studies have conceptualized it in a variety of ways. The most frequently used HRQOL model is based on work by Wilson and Cleary [10]. The Wilson and Cleary model is a causal model that links 5 levels of health outcomes: biological and physiological variables, symptom status, functional status, general health self-perceptions, and overall quality of life [11]. Individual and environmental characteristics influence the symptom status, functional status, general health self-perceptions, and overall quality of life; non-medical factors also have an influence on the overall quality of life [11].

The Wilson and Cleary model was revised in 2005 by Ferrans et al. [12]. In particular, they redefined the influence of individual and environmental characteristics on the health outcomes by adding a link between these factors to biological function (originally “biological and physiological variables”), but maintained the other characteristics. Ferrans et al. also removed unnecessary intermediary linkages, as well as the non-medical domain, because all non-medical factors can be categorized as either individual or environmental [12]. The revision resulted in a simpler and clearer depiction of health outcomes and related factors. A recent systematic review of HRQOL models recommended the revised Ferrans et al. model [12] because it better explains HRQOL than the original Wilson and Cleary model [10].

Previous studies have identified factors associated with the HRQOL of PLWHA. These include age, sex, ethnicity, education level, marital status, employment, social support, duration of living with the infection, CD4+ cell count, HAART treatment status, presence of HIV-related symptoms, co-morbidities, and psychological symptoms [13–18]. Previous HIV/AIDS studies have used various HRQOL instruments, but no instrument is optimal and no single instrument is accepted as the ‘gold standard’ [19]. Furthermore, most instruments used to assess HRQOL in clinical studies of PLWHA do not include indicators of oral health, although it is well-documented that oral mucosal lesions and oral health problems, such as dental caries and periodontal disease, are common in PLWHA [19–24].

Oral diseases may cause symptoms such as pain, discomfort, altered taste, and a burning sensation. Additionally, oral symptoms may interfere with the chewing of food, pronunciation of certain words and sounds, and smiling and socializing with confidence. The Oral Health Impact Profile (OHIP), developed by Slade and Spencer [25], is one of the most widely used instruments to assess the impact of oral diseases on life experiences or oral health-related quality of life (OHRQOL). This instrument is based on the conceptual model for measuring oral health proposed by Locker [26]. Although many studies have assessed the determinants of HRQOL in PLWHA, only few have reported the relationship between OHRQOL and HRQOL.

The purpose of this study was to investigate the association among oral symptoms, OHRQOL, and HRQOL of HIV-infected individuals receiving medical care in Kota Bharu (Kelantan, Malaysia). We used the revised Wilson and Cleary model of HRQOL by Ferrans et al. [12] to examine the relationships of measured health outcomes with their determinants.

## Methods

### Population and sample

This cross-sectional study was conducted from April to May of 2013. The source population comprised of HIV-infected individuals receiving medical care at Hospital Raja Perempuan Zainab II (HRPZ II), Kota Bharu, Kelantan. HRPZ II is a tertiary referral centre for the management of HIV for people living in Kelantan and neighbouring states, particularly Terengganu and Pahang. The Infectious Disease Clinic, under the purview of the Department of Medicine, provides medical care for HIV-infected individuals. Clinical sessions are held twice weekly, and about 20 individuals are seen during each session.

The inclusion criteria were aged 18 years and above, has been living with HIV for at least 1 year, and was able to write and read in the Malay language. The sample size needed to adequately address the study objective was calculated for a power of 80% and significance level of 0.05. Thus, a sample size of 123 was needed. The ethical approval to conduct this study was obtained from the Universiti Sains Malaysia Human Research and Ethics Committee and the Ministry of Health Malaysia Medical Research and Ethics Committee.

### Research tools

The Malay version of short Oral Health Impact Profile (S-OHIP(M)) was used to measure the impact of oral problems on OHRQOL [27]. This questionnaire has 2 items in each of the following 7 domains: functional limitation, physical pain, psychological discomfort, physical disability, psychological disability, social disability, and handicap. A 5-point Likert scale was used to assess the frequency of oral impacts during the previous 12 months, and coded as ‘0’ for ‘never’, ‘1’ for ‘hardly ever’, ‘2’ for ‘occasionally’, ‘3’ for ‘fairly often’ and ‘4’ for ‘very often’. The total score of all 14 items (range: 0 to 56) was calculated to determine the overall severity of the impact, as a reflection of functional status. A higher S-OHIP(M) score indicates greater oral impact, and a poorer OHRQOL. The prevalence of impact which was the percentage of participants who reported ‘fairly often’ or ‘very often’ to one or more items was also determined.

The HRQOL of the participants was measured using the Malay version of the 36-item Medical Outcome Study Short Form (SF-36) [28]. This tool covers 8 health domains, assessed by 35 items: 10 physical functioning items (limitations in physical activities because of health problems), 4 role-physical items (limitations caused by physical health problems), 2 bodily pain items, 5 general health items, 4 vitality items (energy and fatigue), 3 role-emotional items (limitations caused by emotional problems), 2 social functioning items (limitations in social activities because of physical or emotional problems), and 5 mental health items (psychological distress and well-being). An additional item asks about self-perceived current general health status compared to the previous year, which we used to represent the general health perceptions component described in the revised Wilson and Cleary model of HRQOL. Items in the role-physical and role-emotional domains use ‘yes/no’ answers and other items are scored on 3- to 6-point Likert scales. For each domain, the coded and re-coded item scores were summed and transformed according to the standard SF-36 scoring algorithm [29]. The transformed domain scores are reported on a scale of 0 (worst possible health state) to 100 (best possible health state).

**Fig. 1:**
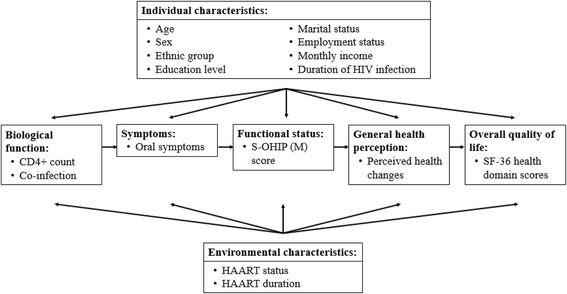
Conceptual framework of variables examined in this study, based on the revised Wilson and Cleary model by Ferrans et al. [12]

Additionally, a structured self-administered questionnaire (Additional file 1) was developed to determine individual characteristics (age, sex, ethnic group, education level, marital status, employment status, monthly income, and duration of HIV infection), environmental characteristics (HAART status and duration), biological function (CD4+ T-lymphocyte count and presence of co-infection), and oral symptoms (toothache, cavitated tooth, gum pain, gum swelling, gum bleeding, gum abscess, loose tooth, bad breath, and mouth ulcer). The content validity of the questionnaire was determined by an expert panel, and a pre-test was given to 30 PLWHA who were receiving care at the Infectious Disease Clinic, Hospital Raja Perempuan Zainab II, Kota Bharu, Kelantan. Participants’ written informed consent was obtained. Figure 1 shows the conceptual framework of variables examined in this study.

### Data collection

A systematic random sampling method was used to identify potential participants from the patient appointment list. The main researcher approached potential participants while they were waiting to be seen by physicians, and invited them to participate in this study. Written informed consent was obtained from each participant prior to enrolment. All questionnaires were self-administered and anonymous. Completed questionnaires were immediately collected.

### Statistical analysis

Data entry and analysis of the results were performed using IBM SPSS for Windows (version 22). All data were first examined and cleaned to prevent errors. Descriptive statistics of all variables were determined; mean and standard deviation (SD) for continuous variables and frequency and percentage for categorical variables. An analysis of variance (ANOVA) was used to compare the mean S-OHIP(M) scores of participants with different general health perceptions. A one sample *t*-test was used to compare the mean SF-36 health domain scores with those of the Malaysian general population, reported previously [30].

The association of S-OHIP(M) score with oral symptoms and with overall quality of life (mean SF-36 health domain score) were determined by univariable and multivariable analysis using simple linear regression and general linear regression analysis, respectively. The multivariable analysis of the association between oral symptoms and S-OHIP(M) score controlled for age, sex, ethnic group, education level, marital status, employment status, monthly income, duration of HIV infection, CD4+ T-lymphocytes count, HAART status and duration, and presence of co-infection. The multivariable analysis of the association between S-OHIP(M) score and SF-36 health domain mean scores controlled for age, sex, ethnic group, education level, marital status, employment status, monthly income, duration of HIV infection, CD4+ T-lymphocytes count, HAART status and duration, presence of co-infection, oral symptoms, and general health perceptions. Variable selection was done using three methods: stepwise selection, backward selection, and forward selection. Following variable selection, all possible two-way interactions, multicollinearity problems, model assumptions, and outliers were checked.

## Results

A total of 123 PLWHA initially enrolled, but 2 patients (1.6%) were dropped, leaving 121 study participants. The 2 excluded patients gave very similar and dubious answers for all S-OHIP(M) items. Table 1 summarises the characteristics of the 121 study participants. The age range was 18 to 57 years-old and the mean age was 38.9 years-old (SD = 6.42). The participants have lived with HIV for an average of 7.4 years (SD = 4.23). The median personal monthly income was 700 Malaysian Ringgit (MYR) (inter-quartile range [IQR] = 925).

**Table 1:** Characteristics of study participants

Variable	Frequency (%)
Age group (years)	
≤ 30	9 (7.4)
31-40	68 (56.2)
41-50	37 (30.6)
> 50	7 (5.8)
Sex	
Female	50 (41.3)
Male	71 (58.7)
Ethnic group	
Malay	111 (91.7)
Others	10 (8.3)
Highest education level	
Primary school	15 (12.4)
Secondary school	94 (77.7)
Certificate/diploma	9 (7.4)
Degree	3 (2.5)
Marital status	
Never been married	31 (25.6)
Married	58 (47.9)
Divorced/widowed	32 (26.4)
Employment status	
Regular jobs	76 (62.8)
Odd jobs	18 (14.9)
Unemployed	27 (22.3)
Personal monthly income (MYR)	
≤ 500	56 (46.3)
501 – 1000	43 (35.5)
1001 – 1500	13 (7.4)
> 1500	9 (7.4)
Duration of HIV infection (years)	
1-5	46 (38.0)
6-10	53 (43.8)
> 10	22 (18.2)
Route of exposure to HIV	
Injecting drug	36 (29.8)
Sexual contact	69 (57.0)
Injecting drug and sexual contact	15 (12.4)
Others	1 (0.8)
CD4+ T-lymphocytes count (cells/mm^3^)	
≤ 199	21 (17.4)
200-499	65 (53.7)
≥ 500	35 (28.9)
HAART treatment status	
No	23 (19.0)
Yes	98 (81.0)
Co-infection	
Hepatitis C	27 (22.3)
Tuberculosis	15 (12.4)

Table 2 shows the self-perceived oral health status of the participants. Most participants had at least one oral symptom (69.4%), and the most common oral symptom was a cavitated tooth (55.4%).

**Table 2:** Self-perceived oral health status of study participants

Variable	Frequency (%)
Perceived oral health status	
Very good	6 (5.0)
Good	59 (48.8)
Fair	37 (30.6)
Poor	16 (13.2)
Very poor	3 (2.5)
Number of oral symptoms	
None	37 (30.6)
One problem	30 (24.8)
Two or more	54 (44.6)
Oral symptoms	
Toothache	33 (27.3)
Cavitated tooth	67 (55.4)
Gum pain	14 (11.6)
Gum swelling	23 (19.0)
Gum bleeding	22 (18.2)
Gum abscess	5 (4.1)
Loose tooth	25 (20.7)
Bad breath	19 (15.7)
Mouth ulcer	9 (7.4)

The prevalence of oral impacts was 33.9%, and the mean S-OHIP(M) score was 8.8 (SD = 7.92). Table 3 shows the mean S-OHIP(M) score for each S-OHIP(M) domain and item, and the proportion of participants reporting impact for each item. The most affected oral health domain was psychological discomfort, which had a mean score of 2.1 (SD = 1.95). An item within the psychological discomfort domain -- discomfort due to food getting stuck between teeth or dentures -- had the highest mean score and prevalence. The lowest oral impact was for social disability, and no participant reported having impacts of ‘fairly often’ or ‘very often’ for “avoided going out”.

**Table 3:** Prevalence and severity of oral impact (S-OHIP(M) score) in study participants

S-OHIP(M) domain and item	Prevalence of impact (%)	Mean S-OHIP(M) score (SD)
Functional limitation	-	1.7 (1.84)
Difficulty chewing any foods	13.2	1.1 (1.19)
Problems caused bad breath	5.0	0.6 (0.94)
Physical Pain	-	1.3 (1.53)
Discomfort eating any food	9.9	0.8 (1.08)
Ulcers in mouth	4.1	0.5 (0.83)
Psychological Discomfort	-	2.1 (1.95)
Discomfort due to food getting stuck	19.8	1.4 (1.23)
Felt shy	9.1	0.7 (1.04)
Physical disability	-	1.5 (1.75)
Avoided eating certain foods	9.9	0.9 (1.11)
Avoided smiling	7.4	0.6 (0.99)
Psychological Disability	-	0.9 (1.48)
Sleep been disturbed	1.7	0.4 (0.75)
Concentration been disturbed	2.5	0.5 (0.86)
Social disability	-	0.5 (0.93)
Avoided going out	0.0	0.2 (0.40)
Problems in carrying out daily activities	1.7	0.3 (0.68)
Handicap	-	0.8 (1.19)
Had to spend a lot of money	0.8	0.3 (0.62)
Felt less confident	4.1	0.6 (0.89)

Table 4 shows the results of multivariable analysis of the association between oral symptoms and S-OHIP(M) score. The S-OHIP(M) score was significantly and positively associated with oral symptoms, particularly toothache, cavitated tooth, gum abscess, and bad breath. Personal monthly income was significantly and negatively associated with S-OHIP(M) score. Overall, the model explains 43.1% of the variation in the S-OHIP(M) score (*R* ^*2*^ = 0.431).

**Table 4:** Association between oral symptoms and severity of oral impact (S-OHIP(M) score) in study participants

Variable	*Adj. b*	95% CI	*t*-statistic	*P* value
Personal monthly income (MYR)	−0.001	−0.003, 0.000	−2.08	0.040
Toothache				
No^a^				
Yes	4.31	1.65, 6.97	3.21	0.040
Cavitated tooth				
No^a^				
Yes	3.44	1.12, 5.76	2.94	0.002
Gingival abscess				
No^a^				
Yes	10.66	5.06, 16.27	3.77	0.004
Bad breath				
No^a^				
Yes	7.59	4.44, 10.74	4.77	<0.001

*Adj. b* = Adjusted regression coefficient

R^2^ = 0.431; the model fits reasonably well; model assumptions were met; there was no interaction between independent variables, and no multicollinearity was detected

^a^Reference category

Descriptive analysis of general health perceptions, as measured by changes in perceived general health status in the past year, indicated that most participants thought they were either somewhat better (40.5%) or much better (22.3%) at the time of the study. However, some participants felt either about the same (28.1%) or somewhat worse (8.3%), and a small number felt much worse (0.8%). An ANOVA that compared the mean S-OHIP(M) scores of participants with different general health perceptions indicated a significant difference (Table 5). A subsequent *post-hoc* test, using Tukey’s HSD procedure, showed that the mean S-OHIP(M) score of participants who thought they were much worse or somewhat worse was significantly different from the score of participants who thought they were about the same.

**Table 5:** Mean S-OHIP(M) scores of participants with different general health perceptions

Variable	Mean S-OHIP(M) score (SD)	*F*-statistic (d.f.)	*P* value
General health perception			
Much/somewhat worse	14.18 (10.08)	3.23 (2, 118)	0.043
About the same	7.38 (7.43)		
Much/somewhat better	8.58 (7.58)		

Table 6 compares the mean SF-36 domain scores of PLWHA in this study (all participants and participants on HAART) with those of the general Malaysian population reported previously [30]. These results show that our study population has a significantly higher score in the bodily pain domain, and significantly lower scores in the physical functioning domain, role-physical domain, general health domain, social functioning domain, and mental health domains. There were no significant differences in the vitality and role-emotional domains. The results were similar when we compared the 98 participants on HAART with the general population.

**Table 6:** Comparison of mean SF-36 domain scores in study participants and the general population of Malaysia

SF-36 domain	Mean score (SD)^a^	95% CI of the difference	*P* value
All participants (*n* = 121)			
Physical functioning	80.79 (20.97)	−8.79, −1.42	0.007
Role-physical	68.18 (40.05)	−21.06, −6.64	<0.001
Bodily pain	77.50 (23.11)	3.38, 11.70	<0.001
General health	59.88 (21.37)	−10.71, −3.02	0.001
Vitality	67.89 (20.76)	−2.63, 4.84	0.560
Role-emotional	74.10 (41.61)	−12.61, 2.36	0.178
Social functioning	79.86 (20.56)	−7.58, −0.17	0.040
Mental health	70.15 (18.04)	−7.76, −1.26	0.007
Participants on HAART (*n* = 98)			
Physical functioning	80.51 (21.00)	−9.68, −1.26	0.011
Role-physical	68.11 (39.12)	−21.76, −6.07	0.001
Bodily pain	76.64 (23.48)	1.98, 11.39	0.006
General health	61.58 (21.09)	−9.39, −0.93	0.017
Vitality	67.40 (20.91)	−3.58, 4.80	0.774
Role-emotional	73.81 (41.52)	−13.74, 2.90	0.199
Social functioning	79.08 (20.29)	−8.72, −0.58	0.026
Mental health	70.98 (18.26)	−7.34, −0.02	0.049

^a^Mean domain scores were compared with the following mean domain scores from a sample of the general population of Malaysia [30]: physical functioning, 85.98 (SD = 17.91); role-physical, 82.03 (SD = 32.12); bodily pain, 69.96 (SD = 17.59); general health, 66.74 (SD = 19.99); vitality, 66.79 (SD = 17.68); role-emotional, 79.23 (SD = 35.92); social functioning, 83.73 (SD = 19.28); mental health, 74.66 (SD = 17.19)

Multivariable analysis of the association between OHRQOL and HRQOL scores shows that participants with lower S-OHIP(M) scores have significantly higher scores in all SF-36 domains (Table 7). There were also several other significant determinants. In particular, sex was significant in the vitality domain, marital status in the physical functioning and role-emotional domains, employment status in the vitality and mental health domains, HAART status in the bodily pain domain, duration of HAART in the vitality and mental health domains, CD4+ T-lymphocyte count in the physical functioning, role-physical, bodily pain, general health, role-emotional and mental health domains, hepatitis C status in the physical functioning, role-emotional, and social functioning domains, tuberculosis in the role-physical and vitality domains, gum swelling in the role-emotional domain, and general health perceptions in the physical functioning, role-physical, bodily pain, general health, and vitality domains.

**Table 7:** Association between S-OHIP(M) score and score in each SF-36 health domain in study participants

Variable	Adjusted regression coefficient							
	PF	RP	BP	GH	VT	RE	SF	MH
Sex								
Female*								
Male	-	-	-	-	−8.45^c^	-	-	-
Marital status								
Never married*								
Married	−11.96^b^	-	-	-	-	20.00^c^	-	-
Divorced/widowed	−9.52^c^	-	-	-	-	12.09	-	-
Employment status								
Regular jobs*								
Odd jobs	-	-	-	-	−2.84	-	-	0.85
Unemployed	-	-	-	-	−9.18^c^	-	-	−7.48^c^
HAART status								
No*								
Yes	-	-	10.32^c^	-	-	-	-	-
Duration of HAART (years)	-	-	**-**	-	−1.06^b^	-	-	−0.61^c^
CD4+ count (cells/mm^3^)								
< 200*								
200-499	11.82^c^	21.21^c^	9.51^c^	7.66	-	22.39^c^	-	8.93^c^
≥ 500	17.39^b^	34.19^a^	15.21^b^	16.63^b^	-	32.70^b^	-	15.18^b^
Hepatitis C								
No*								
Yes	−14.23^b^	**-**	**-**	**-**	**-**	−18.19^c^	−9.71^c^	**-**
Tuberculosis								
No*								
Yes	-	−28.20^b^	-	-	−14.72^b^	-	-	-
Gum swelling								
No*								
Yes	-	-	-	-	-	−18.22^c^	-	-
S-OHIP(M) score	−0.58^b^	−1.85^a^	−1.32^a^	−0.99^a^	−1.02^a^	−1.35^b^	−0.69^b^	−0.81^a^
General health perceptions								
Much/somewhat worse*								
About the same	12.73^c^	30.73^b^	15.24^c^	14.88^c^	10.47	-	-	-
Much/somewhat better	12.65^c^	33.48^b^	18.46^b^	21.29^a^	13.33^c^	-	-	-

*Abbreviations*: *PF* physical functioning; *RP* role-physical; *BP* bodily pain; *GH* general health; *VT* vitality; *RE* role-emotional; *SF* social functioning; *MH* mental health

*Reference category

^a^ *P* < 0.001

^b^ *P* < 0.01

^c^ *P* < 0.05

The adjusted regression coefficient of each model (which explains the variation of the domain score) was as follows: physical functioning, *R* ^*2*^ = 0.290; role-physical, *R* ^*2*^ = 0.465; bodily pain, *R* ^*2*^ = 0.323; general health, *R* ^*2*^ = 0.309; vitality, *R* ^*2*^ = 0.381; role-emotional, *R* ^*2*^ = 0.307; social functioning, *R* ^*2*^ = 0.153; and mental health, *R* ^*2*^ = 0.264. All model assumptions were met, there was no interaction between independent variables, and there was no evidence of multicollinearity.

## Discussion

The prevalence of oral impacts among PLWHA in this study is comparable with that reported for PLWHA receiving medical care in Brazil (34.0%) [31] and HIV-infected individuals receiving care at the Special Need Unit of Adelaide Dental Hospital in Australia (33.3%) [32]. However, the mean severity score of our participants was lower than in these previous studies. The prevalence and severity of oral impacts in the current study were considerably higher than those in the general adult population of Malaysia, reported as 29.3% and 5.87%, respectively [33]. These findings show that the impact of oral diseases is greater for PLWHA than for the general population. The greatest impacts were discomfort due to food getting stuck and difficulty in chewing food. These findings are in accordance with the high number of participants who reported having oral symptoms related to the impacts, namely a cavitated tooth, toothache, and a loose tooth.

About three-quarters of the participants in this study reported having at least one oral symptom, yet most participants perceived their current oral health status as good or very good. Previous studies of different populations in Malaysia reported similar findings. In particular, a sample of antenatal mothers at a teaching hospital in the east coast of Peninsular Malaysia showed that most mothers reported their oral health status as good or very good, although most said they had at least one oral health problem [34]. Another study reported that about half of Malaysian adults (52.4%) perceived their oral health as reasonably good, and 70% were satisfied with their oral health, although almost all of them (98.3%) needed some form of dental treatment [33]. These discrepancies between self–perceived needs and normative needs underline the importance of oral health education to increase oral health awareness in the general public and at-risk groups, such as PLWHA. Our participants also perceived significant oral symptoms, possibly indicating unmet oral health needs among PLWHA in Malaysia. Additional studies also reported self-perceived unmet oral health needs among HIV-positive patients in other populations and countries [35, 36]

According to Sischo and Broder [37], OHRQOL is “a function of various symptoms and experiences and represents the person’s subjective perspective”. In agreement with the original Wilson and Cleary model [11] and the version revised by Ferrans et al. [12], each of which highlighted symptoms as important determinants of functional status, our study showed that participants with oral symptoms (toothache, cavitated tooth, gum abscess, or bad breath) have more severe oral impact. Hence, these individuals had poorer OHRQOL than those without symptoms. These findings are in agreement with previous studies that also showed an association between oral symptoms and OHRQOL [38].

We also found that participants with lower personal income had significantly higher S-OHIP(M) scores, indicating more severe oral impact, in agreement with a study of OHRQOL among PLWHA in Sydney, Australia [39]. Besides income, other studies reported that individual characteristics, such as sex and ethnicity, were significantly associated with the OHRQOL of PLWHA [40]. However, we found no apparent influence of other individual characteristics on the OHRQOL of PLWHA.

Ferrans et al. [12] described environmental characteristics as social and physical environment that can influence health outcomes. In the present study, HAART status and HAART duration represent the environmental characteristics of the participants. HAART, which is currently the most effective treatment for HIV, suppresses viral replication and reduces plasma HIV viral load to below the detection limit, thereby allowing recovery of CD4+ T-lymphocytes and other immunologic functions [41]. HAART was first available in Malaysia in 1996, and starting in 2006, provision of free first-line antiretroviral therapy to all eligible PLWHA has become an important policy of the Ministry of Health as a national response to the HIV epidemic [7]. The introduction of HAART has led to a substantial decline in AIDS-related mortality and morbidity [5, 42–44], including the prevalence of HIV-related oral lesions [45]. However, we found no significant association between HAART and the severity of oral impact among our study participants. This is probably because other oral health problems (such as dental caries), not HIV-related oral lesions, were the main oral health problem among our participants. This is consistent with our finding that a cavitated tooth and toothache were the most common symptoms reported by our study participants, and these were significantly associated with reduced OHRQOL.

Most of our participants were on HAART, had a CD4+ T-lymphocyte count of at least 200 cells/mm^3^, and were under 50 years of age. Hence, it might be expected that their HRQOL would be comparable to that of the general population. However, our results showed that the HRQOL of PLWHA was generally lower than that of the general Malaysian population [30]. Relative to the general population, our participants, including those who were on HAART, had more physical pain and disability, and poorer physical, mental, and social health functioning. These findings clearly indicate that PLWHA have a worse HRQOL. In agreement, a study of more than 3000 PLWHA in the United Kingdom showed that they had a significantly worse HRQOL than the general population, even though most of them had a stable immunologic and virologic status [46]. A study of 521 PLWHA enrolled in the Dublin HIV Cohort study found that the overall HRQOL of the participants was generally comparable with the general Irish population, although their scores in a few HRQOL dimensions were significantly lower [13]. The United States HIV Cost and Services Utilization Study also showed that the HRQOL of symptomatic PLWHA was worse than patients with other chronic diseases [47].

There is limited evidence, particularly from prospective studies, about the benefits of HAART on HRQOL. Some studies showed significant overall improvement under HAART [48, 49], some showed only modest improvement [50, 51], and others raised concerns about deterioration of physical health after long-term HAART [52]. A large multicentre prospective cohort study in the United States demonstrated that HAART was associated with significant short-term improvements in HRQOL, but continued use of HAART did not lead to further changes, in that HRQOL remained declined slightly at a subsequent follow-up [53]. We found that the duration of HAART was positively associated with decreased vitality and mental health scores. The inconsistent findings regarding the effects of HAART on HRQOL may be due to the effect of uncontrolled factors that impact the health and well-being of PLWHA, such as adverse drug reactions [54, 55] and other environmental determinants, such as social stigmatisation and discrimination [56]. Further research is necessary to fully understand the influence of these determinants on the HRQOL of PLWHA.

In this study, we used the SF-36 item that asks about the self-perceived change in health over time as an indicator of the general health perceptions of our participants. This is in accordance with the suggestion by Ferrans et al. [12] that this component is more appropriately measured using a single global question that asks people to rate their general health, instead of using outcome measures that are directed towards one specific dimension of health, such as functioning or symptoms. Clinical research commonly uses global rating of change scales to quantify a patient’s change in health status over time due to an intervention or to follow the clinical course of a condition [57]. Although the outcome may be influenced by various health dimensions, use of a single global question allows the patient to consider the aspects of health that are relevant to him or her, and to give an overall evaluation on a scale ranging from poor to excellent [12]. Some of our participants thought they were much worse or somewhat worse at the time of the survey relative to the previous year, and these participants had significantly higher S-OHIP (M) scores than those who thought they were about the same. These findings provide evidence of an important link between oral health and general health.

Many studies have assessed the determinants of HRQOL in PLWHA, but only few have reported the relationship between OHRQOL and HRQOL. In the present study, we found a significantly positive association between OHRQOL and all HRQOL dimensions measured by the Malay version of the SF-36. These findings are in agreement with a previous study of PLWHA in Minas Gerais, Brazil, which also showed a positive association between OHRQOL and HRQOL [31]. Our study also demonstrated the linkages among HRQOL elements (biological function, symptoms, functional status, and general health self-perceptions) and determinants (individual characteristics and environmental characteristics). This supports application of the revised Wilson and Cleary model by Ferrans et al. [12] for PLWHA.

Most HRQOL instruments used in studies of PLWHA, either generic or HIV-specific, do not have items that adequately assess oral symptoms or oral impacts. Hence, most of the literature that used HRQOL instruments to identify the factors associated with HRQOL among PLWHA failed to capture the influence of oral health [58], although many studies have demonstrated this association in HIV-infected individuals and in other groups of patients and populations [31, 38, 59–61]. Oral health is an important determinant of HRQOL, and should therefore be an integral part of the HRQOL construct used in studies of PLWHA. An OHRQOL instrument, such as OHIP or the Oral Impact of Daily Performance (OIDP), should be used as an adjunct to the currently available HRQOL instruments in future studies.

This study has several limitations. First, all of our participants attended follow-up care at the hospital, and were therefore mostly in relatively good physical condition. Thus, our results cannot be applied to all PLWHA, such as those who are inpatients, those at home and unable to come to the clinic, and those not receiving medical care. Second, the results from a self-administered questionnaire to collect information about oral symptoms must be interpreted with caution, because we used a close-ended response format that lists all possible oral symptoms. Although we included one open-ended question to allow participants to state symptoms other than those listed, none of the participants used this option, so it is possible that we might have missed certain symptoms. Despite this limitation, the use of a self-administered questionnaire provides greater anonymity for study participants. A self-administered questionnaire also allows the participants to take their time in answering questions, without being led by an interviewer.

## Conclusions

Oral health problems are common among PLWHA who receive medical care at the Infectious Disease Clinic, HRPZ II, in Kota Bharu (Kelantan, Malaysia). This finding highlights the importance of collaboration between medical and dental professionals to improve the delivery of oral health care services to PLWHA. Our study also provides evidence for important links among oral symptoms, OHRQOL, and HRQOL in PLWHA. This indicates that measures of oral symptoms and oral impacts should also be used in measuring the HRQOL of PLWHA.

## Additional file

Additional file 1: Structured questionnaire. (PDF 77 kb)

## Abbreviations

CD4: cluster of differentiation 4
HAART: highly active antiretroviral therapy
HIV: human immunodeficiency virus
HRQOL: health-related quality of life
MYR: Malaysian Ringgit
OHRQOL: oral health-related quality of life
PLWHA: people living with HIV/AIDS
SD: standard deviation
SF-36: 36-item Medical Outcome Study Short Form
S-OHIP(M): the Malay version of short Oral Health Impact Profile
VIF: variance inflation factor
