# Supplementary material for: Association among oral symptoms, oral health-related quality of life, and health-related quality of life in a sample of adults living with HIV/AIDS in Malaysia
Source: BMC Oral Health. 2017 Aug 22;17:119. doi: 10.1186/s12903-017-0409-y (PMC5568203; doi:10.1186/s12903-017-0409-y)
Supplement: Additional file 1: — Structured questionnaire. (PDF 77 kb) [file 12903_2017_409_MOESM1_ESM.pdf]

Structured Questionnaire

Please answer all questions

Personal Characteristics

1. Age: …………years

2. Sex:

   Male ☐

   Female ☐

3. Ethnic group:

   Malay ☐

   Chinese ☐

   Indian ☐

   Others ☐ Please state ………………………………..

4. Highest education level:

   No formal education ☐

   Primary/Elementary school ☐

   Secondary school/'O' level ☐

   Vocational training/Certificate ☐

   Diploma/'A' Level ☐

   Tertiary level/Degree ☐

5. Marital status:

   Never been married ☐

   Married ☐

   Divorced ☐

   Widowed ☐

6. Employment status:

   Regular job ☐

   Odd job ☐

   Unemployed ☐

7. Total personal monthly income: RM…………………

   (Please estimate if you do not remember the exact amount)

HIV Status

8. Route of HIV exposure:

   Injecting drug ☐

   Sexual contact ☐

   Others ☐ Please state …………………………

9. Duration of HIV infection: …………years

10. HAART treatment status:

    Yes ☐

    No ☐

11. Duration of HAART treatment: …………years

12. Latest CD4+ cell count (cell/mm^3):

    199 and below ☐

    200-499 ☐

    500 and above ☐

13. Presence of co-infection:

    Hepatitis C ☐ Yes ☐ No

    Tubeculosis ☐ Yes ☐ No

Perceived Oral Health Status

14. In general, how do you rate your current oral health status?

Very good	☐
Good	☐
Fair	☐
Poor	☐
Very poor	☐

15. Do you have any of the following oral health problem?

Toothache	☐ Yes	☐ No
Cavitated tooth	☐ Yes	☐ No
Gum pain	☐ Yes	☐ No
Gum swelling	☐ Yes	☐ No
Gum bleeding	☐ Yes	☐ No
Gum abscess	☐ Yes	☐ No
Loose tooth	☐ Yes	☐ No
Bad breath	☐ Yes	☐ No
Mouth ulcer	☐ Yes	☐ No
Others	☐ Yes. Please state	

THANK YOU
